# The export of hazardous industries in 2015

**DOI:** 10.1186/s12940-016-0091-6

**Published:** 2016-01-19

**Authors:** Barry Castleman

**Affiliations:** PO Box 188, Garrett Park, MD 20896 USA

**Keywords:** Corporate double standards, Corporate social responsibility, Globalization, Suppliers, Distributors

## Abstract

In the 1970s, there were many reports of toxic hazards at corporate subsidiaries in the developing world that were no longer tolerated in the corporations’ “home” countries. Following the chemical disaster in Bhopal, India, in 1984, leading corporations then announced that they applied uniform standards of worker and environmental protection worldwide. With globalization, corporations should also be obliged to take responsibility for their separate supplier, contractor and distributor companies, and licensees of their technology.

The asbestos industry today consists of national corporations. Individual countries must overcome the influence of the asbestos-exporting countries and asbestos companies and stop building with asbestos, as recommended by WHO, ILO, and World Bank. WHO precautions for limiting governmental interaction with the tobacco industry should be applied in dealing with the asbestos industry.

## Background

The environmental movement in some industrialized nations in the 1970s brought unprecedented pressures on the most hazardous and polluting industries. Media coverage and public awareness led to the enactment of laws and the implementation of governmental regulations on chemical and asbestos industries, among others. Companies opposing regulation protested that they would face ruinous competition from unregulated foreign competitors.

But not all the “foreign competition” was foreigners or competitors. All that Amatex, a US asbestos textile company, needed to do to avoid regulation of its manufacturing process, was to move its production just across the Mexican border [1]. Japan’s Kawasaki Steel built a sintering plant in Mindanao, in the Philippines, in the mid-1970s. This steelmaking process produced vast amounts of air pollution including trace metal impurities from the iron ore, coke and iron ore dust, and oxides of sulfur and nitrogen. A Manila newspaper reported that it was a “dirty” industry that could not be built in Japan because of pollution concerns, but “the Philippine authorities have no objection to its installation in the under-polluted southern island.” [1].

In the 1970s, there developed a growing concern about the export of hazardous industries to the developing world and about the responsibility of global corporations for transferring protective safeguards along with the industrial processes, products, and wastes. Today, global corporations still dominate manufacturing and trade, but they often manage this through arrangements with separate companies rather than through their corporate subsidiaries. This complicates the challenge of getting global corporations to improve rather than exploit hazardous conditions in the developing world.

### Before regulation of hazardous US industries caused international flight

Hueper wrote in a 1949 US government monograph that asbestos and a number of chemicals were carcinogenic. He warned that with the growth of industry in the developing world, “The bad record, carcinogenically speaking, which resulted from the hurried development of large-scale chemical industries during and following World War I may be repeated.” He also wrote that, “Criminal codes should take cognizance of the fact that the willful and undue exposure of an individual to a carcinogenic occupational agent for personal gain by another party is for all practical purposes equivalent to an attack with a deadly weapon with a delayed action mechanism.” [2].

Despite the accumulating scientific literature demonstrating the carcinogenicity and other chronic effects of major industrial chemicals, there was minimal regulation of industry anywhere in the world before the 1970s. Trade unions and workers were generally unaware of delayed health effects and the body of knowledge about them. Large corporations employed physicians and industrial hygienists who were aware of the literature, but these health professionals worked for corporate management. They usually had no authority to order improved industrial ventilation or to even tell workers that materials they were exposed to could cause specific forms of cancer.

Even when workers developed occupational diseases, particularly long-delayed conditions such as occupational cancer and pneumoconioses, they rarely became aware of their work as the cause of their disability. In earlier decades and into the 1970s, company doctors examining workers and seeing asbestosis on their chest X-rays failed to tell the workers. It was corporate policy (DuPont, Bethlehem Steel, Johns-Manville) [3].

Under these conditions of virtually no information, no regulation, and no compensation, well-established mortal hazards continued to be widespread and uncontrolled. When industry protested the requirements of a workers’ compensation law in New York in 1935, securing weakening provisions, the companies threatened to move to other states, not other countries [3]. Workers’ compensation laws alone did little to get industry to make expenditures to prevent occupational diseases. Before 1970, there wasn’t much else going on, even to inform, let alone protect US workers from health hazards whose control would involve significant costs.

States in the US avoided creating “barriers to industry.” Some states published in their legal codes the Threshold Limit Values, occupational exposure limits for toxic agents in the workplace air proposed by a volunteer committee of a professional association. But these limits were recommended as good practice guidelines and were not enforced at all by state health and labor officials. Enforcement would have required financing the state agencies to have laboratories capable of doing air sampling and analyses for hundreds of substances -- and empowering and directing state inspectors to issue fines for violations. Just as the corporations were unwilling to compete in safety at the expense of profitability, state governments were unwilling to compete in safety at the expense of losing industries and jobs.

The industrial medicine and hygiene establishment opposed mandatory industrial hygiene codes. At the 1954 meeting of the Industrial Hygiene Foundation, industry lawyer Theodore Waters said administration of workplace limits “in a police sense” would be a “tool in the hands of labor unions.” Harvard professor and corporate consultant Philip Drinker agreed that any codes should be merely “advisory and not mandatory”, otherwise they would be “a severe burden on industry and an unfair one.” None of the company doctors on the panel or in the audience took exception to this view [4].

### Corporate “double standards”

The large-scale export of hazardous industries to the developing world coincided with the global expansion of industry and the modern era of regulation in the US and Europe in the 1970s. Government authorities enforced new laws to regulate airborne exposure to toxic substances at work and pollution in the environment. Most of the new industry set up in the developing world was just the global expansion of business-as-usual, but some was the selective export of ultra-hazardous, discredited technologies. Major “runaway industry” shifts occurred in relatively few cases, such as manufacture of asbestos textiles and benzidine dyes, and the recovery of arsenic as a by-product of smelting certain copper ores [1].

The larger problem was corporate “double standards” in industrial hazards that emerged as industries faced regulation and liability in the most industrialized nations, driven by growing awareness of workers and the public about the disease threat from certain industrial air contaminants and water pollutants. These restrictions were absent in the developing world where rapid industrial expansion was occurring. When challenged, the multinational corporations could come up with no justification for exposing people in some countries to more of these poisons than was allowed in the countries where they were based. They made claims about having no double standards, but these claims were frequently incapable of withstanding scrutiny of the corporate subsidiaries in Asia, Latin America, and Africa [5].

Largely revealed in scattered newspaper and magazine articles, corporate double standards were reported in many countries, in many industrial processes: dye manufacture, numerous asbestos product manufacturing industries, trichlorophenol and chromate manufacture, mercury-cell chlorine manufacture, steelmaking, polyvinyl chloride manufacture, arsenical pesticide manufacture, and polychlorinated biphenyl waste disposal [5]. An International Labor Office study concluded that, in comparing the health and safety performance of home country and foreign subsidiary operations of multinational corporations, “it could be generally said that the home country operations were better than those of the subsidiaries in developing countries” [6].

The United Nations Centre on Transnational Corporations noted in 1985, “[E]xamples of ‘double standards’ in worker and environmental protection have been documented covering a broad range of transnationals, industries and countries” [7]. The Centre, which began in 1974 and closed in 1992, was the UN’s attempt to focus on the global corporations and try (in vain) to develop a code of practice that global corporations might reasonably be expected to follow.

### The Bhopal Gas tragedy

Then came the chemical disaster in Bhopal, India, in December of 1984. In a matter of hours, several thousand people died, many thousands more would succumb later, and well over 100,000 would be permanently disabled. The cause was a massive release of 41 metric tons of volatile methyl isocyanate, a chemical so dangerous that large German and Japanese chemical corporations wouldn’t even store it in large tanks. Relentless media attention disclosed striking contrasts between Union Carbide’s Bhopal plant and another Carbide plant making the same pesticide in West Virginia.

The double standards at Bhopal included numerous vital aspects of design and operation, compounded by management cut-backs in staffing, worker pay, worker training, and maintenance of functional process safeguards (e.g., refrigeration for MIC storage) in the money-losing pesticide plant [8, 9].

The worldwide public reaction to the Bhopal disaster led major chemical corporations to issue global corporate policy statements in the late 1980s that said in various ways, “we have no double standards.” These large companies could thereafter reasonably be expected to demonstrate that the new projects they propose would be up to the highest standards they practice anywhere. Specifically, there are questions a country could justifiably ask a foreign investor before allowing the construction of a new industrial plant (e.g., Guidelines for environmental review of industrial projects evaluated by developing countries) [10].

### Corporate social responsibility elusive in the era of globalization

In recent years, globalization has reduced the visibility of corporate double standards on health, safety, and the environment. Questions of responsibility become easier to shirk in a shifting maze of suppliers, contractors, distributors, and licensees of technology -- all separate companies that are not subsidiaries of the global corporations. Governments in the developing world need to identify the foreign corporations whose business created the basis for an application for plant construction filed by a local entrepreneur. Only then can the prospective host government properly investigate the global corporation’s experience with any hazardous technology involved.

It is tragic that the global chemical and microelectronics corporations neither disclose nor take responsibility for the behavior of these agents of their profitability (suppliers, contractors, distributors, licensees) – in the same way that many of them do for their more easily identifiable subsidiaries.

A “Code of Sustainable Practice in Occupational and Environmental Health and Safety for Corporations” was drafted at a conference on “Dangerous Trade”, with globalization in mind. The Code is an attempt to hold the corporations to the highest standard of toxic substances control they practice anywhere, everywhere; and it applies to all the corporations’ subsidiaries, suppliers, contractors, distributors, and licensees [11].

### Sweatshop safety accord

The devastating collapse of the Rana Plaza building in Bangladesh in 2013 killed 1138 garment workers and injured hundreds more. It occurred only months after the fire at Tazreen Fashion, which caused 112 deaths. As the names of the well-known brands that these Bangladesh clothing makers were supplying became public, global corporations were obliged to create and agree to the Bangladesh Accord on Fire and Building Safety.

The Accord began regular inspections of buildings for fire, electrical, and structural integrity in February 2014. The findings are published, including photographs of hazardous conditions, on the Accord’s website www.bangladeshaccord.org The Accord will conduct independent inspections of 1800 factories. More than 150 global brands and retailers have signed the Accord. The Accord requires brands to pay factories enough to assure safety, including paying the cost of repairs immediately after an inspection reveals hazardous conditions. While buildings are closed for the repairs, the workers are paid. International and Bangladesh unions are actively involved in the Accord, as is the International Labor Organization [12]. Walmart and Gap Inc. declined to sign the legally-binding Accord, opting instead to pursue “voluntary” programs.

### Asbestos

As the result of social movements around the world, asbestos is banned in over 50 countries. Yet most of the world’s people live in countries where asbestos is still used, often with few if any protective measures. Global consumption since the turn of the new century has remained at about 2 million tons annually, as some countries banned asbestos and others increased their use of it. Over 90% of the asbestos is used in asbestos-cement sheets and pipes, most for asbestos-cement roofing. Over 85% of world asbestos consumption is in Asia.

The World Health Organization has called for a global ban of all forms of asbestos and estimated the annual death toll worldwide at more than 107,000 due to lung cancer, mesothelioma, and asbestosis from occupational exposure [13]. Many thousands more die from environmental, non-occupational cancer caused by asbestos, including the family members in workers’ households exposed to dust brought home on the workers’ clothes. Yet others die from other cancers caused by asbestos -- including laryngeal and ovarian cancer, according to the International Agency for Research on Cancer [14].

Global corporations can play a constructive role where their corporate standards are superior to what may be locally required of them. Companies including Unilever, Dow, and ICI, like the World Bank [15], have announced that they have global policies of 1) not using asbestos materials in constructing new facilities and 2) observing procedural safeguards when asbestos materials have to be disturbed or demolished in existing facilities. Public disclosure of the texts of these corporate policies would be a useful spur to national companies to do likewise. Such disclosure would help expand the market for alternatives to asbestos-cement roofing, increasing demand and lowering the price of safer substitutes while supporting the case for banning asbestos in the country. However, officials with ICI, Unilever and other global corporations have been reluctant to disclose the texts of their policies.

Back in the 1970s, many asbestos companies in the developing world were subsidiaries and customers of global asbestos corporations. But in the new century, there are no corporate asbestos giants remaining. The industry today consists mainly of national companies in each country, though there may occasionally be some ownership by foreign companies from asbestos-mining countries (mainly Russia and Kazakhstan). The involvement of Russian and Kazakh asbestos interests in Vietnam may play an important role in the opposition to banning asbestos there.

The asbestos companies of today are run by businessmen who stayed in or got in when the multinational corporations got out. This industry spends millions hiring scientists to write and publish “downright dishonest science” in the literature [16]. The asbestos companies worldwide are well connected and use the same propaganda and strategies everywhere to minimize their costs for disease prevention and compensation. In recent years, doctors raising concerns about asbestos in India, Thailand, and Brazil have been threatened with legal action by the asbestos industry.

A multibillionaire asbestos owner-executive, Stephan Schmidheiny, was sentenced to 18 years in jail for creating an environmental disaster causing thousands of deaths from mesothelioma by an Italian trial court and court of appeal; he only avoided conviction when the highest court threw the case out on a legal technicality (crimes prosecuted after the statute of limitations had run) [17]. A murder case against Schmidheiny is poised to proceed in 2016, pending a ruling from the Italian constitutional court.

No other industry has a comparable record of documented bad practices in occupational and environmental health. In the US, decades of litigation over asbestos injury compensation have pried loose a vast number of internal documents from the asbestos companies. These corporate documents reveal a veritable encyclopedia of menacing business practices. The history unearthed has involved the asbestos industry worldwide, going back to the 1920s.

This record includes [3, 18, 19]:

- the suppression of medical and experimental findings
- manipulation of published reports
- suppression of reference to asbestos hazards in the trade press
- publication of statements and reports by trade associations that asbestos products are not toxic
- withholding of information on asbestos disease from governmental authorities
- prolonged violation after regulations required health warning labels on asbestos products
- marketing of products without warning labels in some countries after starting to affix warnings on the same products in other countries
- targeting of doctors raising public awareness about asbestos hazards
- settlement of damage suits on condition that the lawyer representing the workers file no more such cases
- non-disclosure to employees of asbestosis revealed in their medical examinations
- firing workers and busting unions for protesting asbestos hazards
- firing and replacing workers before they had time to develop asbestos diseases from their exposures
- exporting banned asbestos products,
- labeling asbestos-containing products “asbestos-free”
- removing the word “asbestos” in advertising asbestos products bearing no warnings
- selling asbestos for use in children’s modeling compounds
- sub-contracting of hazardous asbestos maintenance work
- wanton disposal of wastes around asbestos factories
- prolonged failure to take basic sanitary precautions to keep workers from taking asbestos dust home to their families on their clothes

In the new century, asbestos interests have spent many millions of dollars contracting and publishing “product defense” articles to exonerate chrysotile asbestos, the type of asbestos that accounted for 95 % of world asbestos use in the 20^th^ century and the only type of asbestos in international trade since then [20, 21]. Governments with close relations with asbestos interests have repeatedly blocked including chrysotile asbestos under the Rotterdam Convention’s prior-informed-consent warning requirements for widely-banned, hazardous substances in international trade.

The asbestos industry is making profits from selling and exporting a deadly product, and in addition to the human tragedy it is creating, the industry is placing on the shoulders of individuals, their families, their communities and their governments the enormous economic costs of ill health, mortality and contaminated environments. The asbestos industry should accordingly be treated by governments the way the World Health Organization recommends governments treat the tobacco industry.

### Treating the asbestos industry like the tobacco industry

The WHO Framework Convention on Tobacco Control recognizes the “need to be alert to any efforts by the tobacco industry to undermine or subvert tobacco control efforts” and to take action “to protect (tobacco control) policies from commercial or other vested interests of the tobacco industry”[22]. This is based on guiding principles including Principle 1: “There is a fundamental and irreconcilable conflict between the tobacco interests and public health policy interests.” To implement this, WHO recommends that governments “should interact with the tobacco industry only when and to the extent strictly necessary to enable them to effectively regulate the tobacco industry and tobacco products” [22].

Because of the asbestos industry’s parallel history of subverting public health policy, similar limitations should be followed by governments making policy decisions on asbestos in interacting with the asbestos industry’s representatives. Governments should not feel obliged to put up with endless lobbying, delaying tactics and arguments about “controlled use” of the killer dust. This is an industry whose very survival depends on minimizing the expense of prevention and compensation, mediated through links with politicians, government officials, media, the courts, doctors, medical institutions, universities, and unionists. Governments should interact with the asbestos industry only to the extent necessary to ban new asbestos product use as quickly as possible and to provide protective measures for dealing with asbestos products already in place.

## Conclusion

The export of hazardous industries to the developing world is still largely a responsibility of the global corporations that dominate world trade, and increasingly so with international trade agreements enabling these giants to increase their presence in markets worldwide. The subcontracting of hazardous work has gone global, with the expansion of hazardous industries, particularly in Asian countries. Hazardous and polluting industries operate in collapsing buildings with no safety provisions, untouched by regulation and liability. If disaster occurs, it takes an investigation even to determine which foreign corporations were the customers for the products the deadly plants were making.

Corporate leaders need to own up to their responsibilities by publicly disclosing the identities of their suppliers, in particular, and accepting the responsibility for assuring that suppliers follow the highest standards of worker and environmental protection -- just as the corporations do for clearly identifiable corporate subsidiaries. Non-governmental organizations, governments, and international bodies can take measures to increase transparency and disclosure of business connections between the giant corporations and their affiliates, particularly when tragedies occur.

One industry stands out as a discredited, hazardous industry that just won’t go away quietly: asbestos. The last global asbestos company was the French Saint-Gobain, which decided to sell its Brazilian asbestos mine and convert its asbestos-cement plant there to non-asbestos fiber-cement in 1999. But when the global companies got out, others were there to take over, and Saint-Gobain’s Brazilian subsidiary faces stiff competition from national asbestos companies in the states of Brazil where asbestos has not yet been banned. Similarly, asbestos businesses started in the past century by foreign corporations have been taken over by local entrepreneurs in India and many other countries. National governments need to overcome the obstruction of the asbestos industry and act on the knowledge that, as the WHO puts it, “the most efficient way to eliminate asbestos-related diseases is to stop the use of all types of asbestos” [23].
